# IMPACT OF COVID-19 MITIGATION POLICIES: LESSONS LEARNED FROM US AND MIDDLE-HIGH INCOME COUNTRIES

**DOI:** 10.1093/geroni/igac059.030

**Published:** 2022-12-20

**Authors:** Preeti Zanwar, K J Vinod Joseph, Zaliha Omar, Flavia Santos, Evguenii Zazdravnykh, Patricia Heyn, Arokiasamy Perianayagam

**Affiliations:** Jefferson College of Population Health, Philadelphia, Pennsylvania, United States; International Institute for Population Sciences, Mumbai, Maharashtra, India; School of Health Sciences, Fujita Health University, Toyoake, Aichi, Japan; University College Dublin, Dublin, Dublin, Ireland; National Reserach University, International Center of Health Economics, Policy, and Management, St. Petersburg, Leningrad, Russia; Center for Optimal Aging, Marymount University, Fairfax Station, Virginia, United States; National Council of Applied Economic Research, New Delhi, Delhi, India

## Abstract

The COVID-19 pandemic has been a natural global epidemiological experiment unique to our century and a massive shock to older adults and to systems that care for them. There was a lack of a global unified plan to mitigate and control the spread of COVID-19. Several middle-or-high income nations struggled to control the viral spread resulting in increased mortality due to a combination of lack of public health measures and existing disparities which were magnified during the pandemic. The purpose of this review by a team of international experts is to (1) to examine reasons for the varied COVID-19 responses within U.S. and among other middle-or-high-income countries and the emergence of variants and vaccine inequities, and (2) to examine the country specific burden of cultural/structural/political determinants on access to care and mortality among older adults in various settings.

